# A Delphi consultation to assess indicators of readiness to provide quality health facility-based lymphoedema management services

**DOI:** 10.1371/journal.pntd.0006699

**Published:** 2018-09-18

**Authors:** Victoria L. Walsh, LeAnne M. Fox, Molly Brady, Jonathan King, Caitlin M. Worrell

**Affiliations:** 1 Parasitic Diseases Branch, Division of Parasitic Diseases and Malaria, Center for Global Health, Centers for Disease Control and Prevention, Atlanta, Georgia, United States of America; 2 RTI International, Department of Global Health, Washington, DC, United States of America; 3 Department of Control of Neglected Tropical Diseases, World Health Organization, Geneva, Switzerland; University Hospital Bonn, GERMANY

## Abstract

**Background:**

The World Health Organization (WHO) in collaboration with partners is developing a toolkit of resources to guide lymphatic filariasis (LF) morbidity management and disability prevention (MMDP) implementation and evaluation. Direct health facility inspection is the preferred method for documenting the readiness of a country programme to provide quality lymphoedema management services, one of the three MMDP criteria used to demonstrate the elimination of LF as a public health problem.

**Methodology/Principal findings:**

As component of tool development, a Delphi consultation was implemented to gain consensus on six proposed domains and fourteen proposed tracer indicators to measure national programme readiness to provide quality health facility-based lymphoedema management services. A seven-point Likert-type scale was used to rank the importance of proposed domains and tracer indicators. Consensus for inclusion of the indicator was defined *a priori* as 70% or more of respondents ranking the proposed indicator in the top three tiers (5–7). Purposive sampling was used to select 43 representative experts including country representatives, programme implementers, and technical experts. A 55.8% response rate (n = 24) was achieved for the survey. Analysis of the responses demonstrated that consensus for inclusion had been reached for all proposed domains including trained staff (mean = 6.9, standard deviation (SD) = 0.34), case management and education materials (mean = 6.1, SD = 0.65), water infrastructure (mean = 6.3, SD = 0.81), medicines and commodities (mean = 6.3, SD = 0.69), patient tracking system (mean = 6.3, SD = 0.85), and staff knowledge (mean = 6.5, SD = 0.66).

**Significance:**

The Delphi consultation provided an efficient and structured method for gaining consensus among lymphatic filariasis experts around key lymphoedema management quality indicators. The results from this analysis were used to refine the indicators included within the direct inspection protocol tool to ensure its ability to assess health facility readiness to provide quality lymphoedema management services.

## Author summary

Prior to this assessment, there was a need for clearly defined, measurable indicators for global lymphoedema management programmes to use to evaluate their lymphoedema morbidity management and disability prevention services. The results presented in this report outline a framework for indicators to assess healthcare facility-based readiness to provide quality lymphoedema management services. We describe our use of the Delphi methodology to obtain consensus on programme evaluation metrics as a model for elimination or control metrics and targets. The quality indicators developed from this Delphi consultation will be used to improve the direct inspection protocol tool that can be used to assess health facility readiness to provide quality lymphoedema management services LF endemic countries.

## Introduction

Lymphatic filariasis (LF) is a parasitic infection caused by filarial nematodes that are transmitted by mosquitoes. Chronic infection with LF can lead to clinical manifestations such as lymphoedema and hydrocele that have significant impacts on the mobility and quality of life of affected individuals. Further, individuals with lymphoedema are prone to painful and debilitating secondary bacterial infections, known as acute attacks, that are associated with diminished quality of life and progression of disease [1–4]. Approximately 947 million people are at risk for LF in more than 73 countries worldwide [5]. In an effort to reduce suffering, LF has been targeted for elimination as a public health problem by 2020 following the World Health Assembly Resolution 50.29 [6]. The Global Programme to Eliminate LF (GPELF) has established a two-pillar strategy for elimination: (1) interruption of transmission through mass drug administration (MDA) and (2) alleviating the suffering of individuals affected by the chronic manifestations of LF infection through the provision of morbidity management and disability prevention (MMDP) services. To meet the criteria established by the Word Health Organization (WHO) for the MMDP pillar for elimination, national LF elimination programmes are asked to provide data on the number of patients with lymphoedema (or elephantiasis) and hydrocele, the number of health facilities designated to provide care, and the readiness and quality of the care provided [7]. Quality of care assessments can be used to understand what resources are needed to improve services as well as advocate for other sectors or departments within the Ministry of Health to supplement these services.

While the implementation and evaluation activities for MDA have been clearly defined, there is a need for clearer guidance on the provision and assessment of MMDP services for national LF elimination programmes. In order to meet this need, WHO is developing a toolkit to guide LF MMDP implementation and evaluation. One component of the toolkit is a direct inspection protocol, a tool designed to measure readiness to provide quality health facility-based lymphoedema management services in accordance with WHO recommendations. Here, we summarize an expert consultation following the Delphi methodology to reach consensus on domains and indicators that should be used to evaluate health-facility readiness to provide quality lymphoedema management services [10]. The results of the Delphi consultation informed the refinement of the direct inspection protocol tool and will assist national LF elimination programmes demonstrating that they have achieved the requirements for validation of elimination of LF as a public health problem.

## Methods

### Delphi methodology

#### Indicator development

**Fig 1:**
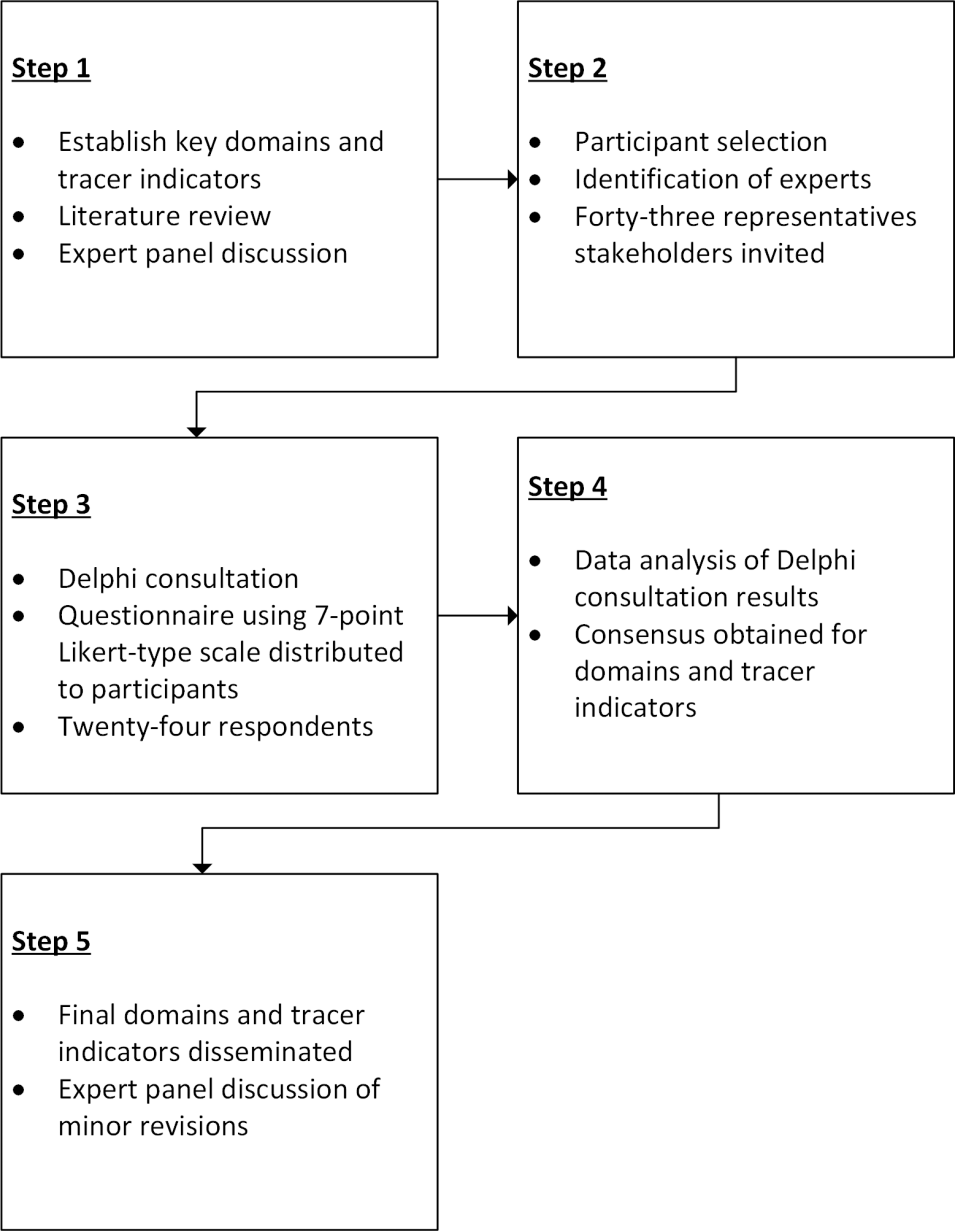
Process of selection of domains and indicators to determine readiness of health facilities to provide quality lymphoedema management services.

To establish consensus on indicators to assess the quality of MMDP services, a Delphi methodology was implemented. The Delphi methodology has been utilized by others in the neglected tropical disease (NTD) field to obtain consensus on programmatic targets as well as indicators for programme monitoring and evaluation [8, 9]. It is a quantitative mechanism to gain consensus on a particular topic among a panel of subject matter experts [10]. The Delphi methodology that we implemented (Fig 1) was based on a framework proposed by Deribe and colleagues in the context of establishing indicators to assess endemicity, elimination, and clinical outcomes of podoconiosis [9, 11].

Based on a literature review, an expert panel proposed six key domains of facility readiness and 14 tracer indicators to measure readiness of health facilities to provide quality MMDP services for lymphoedema care. The six domains selected were modeled after the Service Availability and Readiness Assessment (SARA), a WHO tool for evaluating health facilities, and included availability of: trained staff, case management and education materials, water infrastructure, medicines and commodities, patient tracking system, and staff knowledge [12]. Fourteen tracer indicators were proposed to evaluate the domains (Table 1).

**Table 1:** Proposed quality domains and corresponding tracer indicators.

Domains	Tracer Indicators
1. Trained staff	1. at least one health facility staff member trained in lymphoedema management in the last two years;
2. Case management and education materials	2. at least one guideline for lymphoedema management is present at the health facility; 3. at least one information, education, and communication awareness material for lymphoedema management is present at the health facility;
3. Water infrastructure	4. the main water for the facility is an improved source, is located on the premises, and is functional at the time of the visit;
4. Medicines and commodities	5. antiseptics are present at the facility; 6. antifungals are present at the facility; 7. antibiotics are present at the facility; 8. analgesics are present at the facility; 9. at least one supply for lymphoedema and acute attack management is available at the health facility;
5. Patient tracking system	10. a system for patient tracking with at least one patient recorded in the last 12 months;
6. Staff knowledge	11. clinic staff member able to correctly identify at least one sign or symptom of lymphoedema; 12. clinic staff member able to correctly identify at least one lymphoedema management strategy; 13. clinic staff member able to correctly identify at least one sign or symptom of an acute attack; 14. clinic staff member able to correctly identify at least one strategy to treat a patient with an acute attack

#### Participant selection

Forty-three experts in LF and NTDs representing various stakeholders globally were identified to participate in this consultation (Table 2). This target was determined assuming a 20% loss to follow-up over two steps and aimed to achieve 15 to 25 final participants. The process for selecting these participants was modeled on the recommendations for participant selection outlined in previous literature utilizing the Delphi methodology [10, 13]. Participants were from more than ten countries across all WHO regions with LF-endemic countries and represented country programmes, non-governmental organizations, bi-lateral and multi-lateral organizations, donor organizations, and academic institutions.

**Table 2:** Delphi panel composition (N = 24).

Characteristic	N	(%)
Profession		
Academic/Researcher	8	33.3
Country NTD Programme Representative	4	16.7
Donor Organization Representative	3	12.5
Non-governmental Organization (NGO) Representative	4	16.7
Multilateral or Bilateral Organization Representative	2	8.3
Other	3	12.5
Education/Background		
Biomedical/epidemiology	10	41.7
Clinical	13	54.2
Social sciences	1	4.2
Area of Expertise		
Lymphatic filariasis as well as other NTDs	14	58.3
Lymphatic filariasis	5	20.8
Lymphoedema/lymphoedema management	3	12.5
Hydrocele management	2	8.3
Years of Expertise		
1–4 years	3	12.5
5–9 years	4	16.7
10–24 years	9	37.5
25+ years	8	33.3

#### Questionnaire

Participants were invited by email to participate in the consultation via a link where they could access an online questionnaire. Participants were asked to report demographic information including professional role, educational background, area of expertise, and years of experience in their respective area of expertise. Participants were then asked to evaluate the domains and corresponding indicators using a seven-point Likert-type scale to optimize discriminating power [14, 15]. Finally, participants were asked to rank the importance of each of the domains in determining quality of MMDP services a health facility could provide, ranging from “1 = not at all important” to “7 = extremely important”. Participants were asked to rank how well each of the indicators evaluated the respective domain ranging from “1 = strongly disagree” to “7 = strongly agree”. Participants were also invited to provide open-ended feedback on the domains and indicators.

Seven indicators measured general health facility readiness and quality (e.g. water infrastructure, provision of medications and commodities) and seven measured lymphoedema-specific readiness and quality of services provided by health facilities (e.g. staff training and knowledge). In an effort to harmonize and integrate with ongoing WHO initiatives, it was determined that if the water infrastructure domain was deemed relevant, the tracer indicators for water infrastructure outlined by WHO’s water, sanitation, and hygiene (WASH) in health-care facilities initiative also would be used in the direct inspection protocol for consistency. Therefore, the water infrastructure tracer indicator was not evaluated, thus only thirteen indicators were evaluated in the Delphi methodology versus the total 14 indicators that would be assessed as a component of the direct inspection protocol.

#### Data analysis

Data were analyzed using Microsoft Excel and SAS 9.3 (Cary, NC). Consensus criteria for each domain and tracer indicator were defined *a priori* as follows: consensus for inclusion was achieved if ≥70% of participants ranked the item in the top three categories (5–7); consensus for exclusion was achieved if ≥70% of participants ranked the item in the bottom three categories (1–3); and no consensus was achieved if neither of the above conditions were met.

Counts of respondents’ selections were used to calculate the frequency of selected answers to inform whether consensus had been reached. In addition, the sample mean, median, and range were calculated to assess central tendency for each tracer indicator and domain and to characterize the responses. Central tendency was included in the assessment as a secondary measure of consensus among the respondents. If consensus was not achieved by the above criteria, further refinement and evaluation of the domains and indicators in subsequent rounds of questionnaires was planned until consensus was reached.

As a sensitivity analysis, we evaluated the impact of using more stringent consensus criteria as follows: consensus for inclusion was achieved if ≥70% of participants ranked the item in the top two categories (6–7); consensus for exclusion was achieved if ≥70% of participants ranked the item in the bottom two categories (1–2); and no consensus was achieved if neither of the above conditions were met.

## Results

The response rate for the online survey component of the Delphi consultation was 55.8% (n = 24). The individuals who participated in the survey represented a range of professions (Table 2). A third of participants (n = 8, 33.3%) had more than 25 years of experience in their respective field.

Participants’ responses to the domains and tracer indicators are presented in Tables 3 and 4. In the first round, there was consensus that all six domains of readiness and quality of MMDP services in health care facilities were important. None of the respondents ranked the domains in the bottom three categories. The strongest agreement was observed for trained staff and staff knowledge with 87.5% and 62.5% of respondents respectively indicating they felt that these domains were extremely important. Furthermore, consensus was reached for all thirteen evaluated tracer indicators, though a wider range of ranking was observed. The strongest consensus was observed for tracer indicators related to medicines and commodities—primarily the availability of medicines—as well as the tracer indicators for case management and education materials.

**Table 3:** Participant response to the question: “How important are the following domains in determining quality of MMDP services a health facility could provide?”, n = 24.

	1: Not at all	2: Low	3: Slightly	4: Neutral	5: Moderately	6: Very	7: Extremely	Central Tendency		Consensus top three responses
	N (%)	N (%)	N (%)	N (%)	N (%)	N (%)	N (%)			N (%)
Theme								Mean (SD)	Median (Q1, Q3)	
Trained Staff	-	-	-	-	-	3 (12.5%)	21 (87.5%)	6.9 (0.34)	7 (7,7)	24 (100)
Case management and Education Materials	-	-	-	-	4 (16.7%)	14 (58.3%)	6 (25.0%)	6.1 (0.65)	6 (6, 6.5)	24 (100)
Water Infrastructure	-	-	-	-	5 (20.8%)	7 (29.2%)	12 (50.0%)	6.3 (0.81)	6.5 (6,7)	24 (100)
Medicines and Commodities	-	-	-	-	3 (12.5%)	11 (45.8%)	10 (41.7%)	6.3 (0.69)	6 (6,7)	24 (100)
Patient Tracking System	-	-	-	1 (4.2%)	3 (12.5%)	9 (37.5%)	11 (45.8%)	6.3 (0.85)	6 (6,7)	23 (95.8)
Staff Knowledge	-	-	-	-	2 (8.3%)	7 (29.2%)	15 (62.5%)	6.5 (0.66)	7 (6,7)	24 (100)

**Table 4:** Participant response to the question: “The following indicator adequately evaluates the domain ________.” n = 24.

	1-Strongly Disagree	2-Disagree	3-Somewhat Disagree	4-Neither Agree nor Disagree	5-Somewhat Agree	6-Agree	7-Strongly Agree	Central Tendency		Consensus top three responses
Indicators	N (%)	N (%)	N (%)	N (%)	N (%)	N (%)	N (%)	Mean	Median (Q1, Q3)	%
								(SD)		
**Trained Staff**										
At least 1 facility staff member trained in lymphoedema management in the last 2 years		2 (8.3%)	2 (8.3%)	2 (8.3%)	2 (8.3%)	12 (50.0%)	4 (16.7%)	5.3 (1.5)	6 (4.5,6)	75.0
**Case Management & Education Materials**										
At least 1 guideline for lymphoedema management is present at the facility				1 (4.2%)	2 (8.3%)	13 (54.2%)	7 (29.2%)	6.1 (0.76)	6 (6,7)	91.7
At least 1 IEC awareness material for lymphoedema management is present at the facility					3 (12.5%)	14 (58.3%)	7 (29.2%)	6.2 (0.64)	6 (6,7)	100.0
**Medicines and Commodities**										
Antiseptics are available at the facility				1 (4.2%)	2 (8.3%)	10 (41.7%)	11 (45.8%)	6.3 (0.81)	6 (6,7)	95.8
Antifungals are present at the facility				1 (4.2%)	1 (4.2%)	10 (41.7%)	12 (50.0%)	6.4 (0.77)	6.5 (6,7)	95.9
Antibiotics are present at the facility		1 (4.2%)		1 (4.2%)	1 (4.2%)	10 (41.7%)	11 (45.8%)	6.2 (1.2)	6 (6,7)	91.7
Analgesics are present at the facility				1 (4.2%)	1 (4.2%)	10 (41.7%)	12 (50.0%)	6.4 (0.77)	6.5 (6,7)	95.9
At least 1 supply for lymphoedema and acute attack management is present at the facility					1 (4.2%)	8 (33.3%)	15 (62.5%)	6.6 (0.58)	7 (6,7)	100.0
**Patient Tracking System**										
At least 1 patient recorded in the reporting system in the last 12 months		3 (12.5%)		3 (12.5%)	5 (20.8%)	9 (37.5%)	3 (12.5%)	5.1 (1.5)	6 (4,6)	70.8
**Staff Knowledge**										
Clinic staff member able to correctly identify at least 1 sign or symptom of lymphoedema		2 (8.3%)			2 (8.3%)	8 (33.3%)	12 (50.0%)	6.1 (1.4)	6.5 (6,7)	91.6
Clinic staff member able to correctly identify at least 1 lymphoedema management strategy		2 (8.3%)		1 (4.2%)	3 (12.5%)	7 (29.2%)	11 (45.8%)	5.9 (1.5)	6 (5.5,7)	87.5
Clinic staff member able to correctly identify at least 1 sign or symptom of an acute attack		2 (8.3%)	1 (4.2%)	1 (4.2%)	1 (4.2%)	8 (33.3%)	11 (45.8%)	5.9 (1.6)	6 (6,7)	83.3
Clinic staff member able to correctly identify at least 1 strategy to treat a patient with an acute attack		2 (8.3%)	1 (4.2%)	1 (4.2%)	1 (4.2%)	7 (29.2%)	12 (50.0%)	5.9 (1.6)	6.5 (6,7)	83.4

Based on the sensitivity analysis using stricter criteria for consensus, all domains met consensus criteria under stricter conditions with between 79.2% and 100% of respondents ranking the domains in the top two categories. All of the tracer indicators met the stricter consensus criteria except for staff training in the last two years (66.7%) and at least one patient with lymphoedema recorded in the reporting system in the last 12 months (50.0%).

Common themes from the qualitative feedback included: the need for a more robust definition of training and refresher training, the importance of clinic staff being able to identify more than one sign, symptom, and management strategy, and a need for a more clearly stated definition for a patient tracking system.

## Discussion

The World Health Assembly resolution to eliminate LF was built on a desire to mitigate the harm caused by LF, both by preventing future infection as well as by alleviating the suffering experienced by individuals who present with clinical manifestation as a result of infection [6]. Since the clinical sequelae of LF develop many years after infection and are chronic, national LF programmes must work closely within the health care system to ensure that MMDP services are well integrated, available, and sustainable. While the components of a minimum package of care for lymphoedema and hydrocele patients has been clearly defined [9], there is a need for standardization in the evaluating and reporting of the availability and quality of MMDP services in the provision of the minimum package of care at healthcare facilities. This Delphi consultation allowed input from multiple stakeholders and improved the practicality and acceptability of a standard survey for direct inspection of health facilities to assess readiness and quality of MMDP. Based on previous literature, framing questions using a Likert-type scale for Delphi consultations facilitates straightforward statistical analysis to assess for consensus across respondents [13, 14, 16].

A strength of the Delphi methodology is that it allows stakeholders from a variety of perspectives to offer their expert opinion on the key elements that need to be included in an evaluation of quality services. Using a Delphi consultation, we were able to gather input for indicator development from a range of stakeholders. Our hope is that this approach will lead to broader stakeholder support and acceptability. Based on the diversity of participants, we feel that the consensus achieved reflects the priorities of global partners working towards elimination of LF as a public health problem. However, due to limitations in accessibility we were unable to include the perspectives of two important stakeholders: health facility level staff and affected patients. Steps were taken to include feedback from staff and patients during the pilot testing of the direct inspectional protocol as discussed later.

Though consensus was reached for the domains and indicators, through open-ended feedback experts proposed more stringent criteria to strengthen the indicators to measure the readiness of health facilities to provide quality lymphoedema management care. Citing the critical need for appropriate identification of lymphoedema in patients, experts suggested that a greater emphasis should be placed on the evaluation of staff knowledge. To address this, questions assessing staff knowledge were modified to require two correct responses instead of one for each tracer indicator. No significant changes were made to the components of the remaining domains and indicators.

The fourteen tracer indicators, refined as part of the Delphi consultation, are intended to comprise the questionnaire component of a health facility inspection tool, allowing LF programmes to evaluate the readiness of health facilities to provide quality lymphoedema management services as a component of MMDP programmes. The inspection comprises a facility walkthrough and interview with key health personnel at randomly selected health facilities providing lymphoedema management services. The surveyor evaluates if the facility meets the criteria for each indicator, through direct observation where relevant (e.g. the presence of medicines and commodities). The results of the questionnaire generate a health facility score, by which the programme can evaluate highly performing and poorly performing health facilities. Programmes can also evaluate indicator scores across facilities to evaluate systematic strengths and weakness, in order to implement informed process-improvement steps to strengthen the quality of lymphoedema management services. In addition, the standardization offered through these tracer indicators provides programmes with the ability to compare lymphoedema management services across settings.

We recognize that while the primary focus of this Delphi consultation was assessing lymphoedema management services, hydrocele care is also important in LF endemic countries. We feel confident that we could replicate similar procedures to develop indicators to assess health facility readiness to provide quality hydrocele care. Due to the unique components of care required for hydrocele patients, expert consultation with urologists with expertise in hydrocele management will be conducted to determine the domains and tracer indicators for evaluating readiness and quality of health-facility based hydrocele care. A standardized protocol, the WHO Surgical Assessment Tool (SAT), provides information on general surgical capacity including the availability of hydrocelectomy and is under revision. Efforts are under consideration to include a module specifically evaluating the readiness and quality of hydrocele care.

By demonstrating that the global community is in agreement about the components of lymphoedema management that healthcare facilities must be prepared to provide their patients, there is evidence to support the inclusion of the direct inspection protocol tool in the MMDP toolkit for countries implementing the MMDP component of the GPELF strategy. In an effort to assess the measurability of these indicators in healthcare facilities, the quality domains and tracer indicators identified were included in a pilot direct inspection tool to assess the readiness of healthcare facilities to provide quality lymphoedema management services in Mali, Vietnam, and Haiti [17]. The pilot demonstrated that these indicators were feasible to implement and yielded useful information about the quality of services; however minor changes were incorporated to the survey based on the results of the pilot. The direct inspection protocol is intended to supplement SARA assessments, provide more detailed information on lymphoedema management services, and to ensure programme managers have the information needed to plan for services to meet the needs of individuals with LF.
